# Healthy vs. Unhealthy Food Images: Image Classification of Twitter Images

**DOI:** 10.3390/ijerph19020923

**Published:** 2022-01-14

**Authors:** Tejaswini Oduru, Alexis Jordan, Albert Park

**Affiliations:** Department of Software and Information, Systems College of Computing and Informatics, University of North Carolina Charlotte, Charlotte, NC 28223, USA; toduru@uncc.edu (T.O.); ajorda50@uncc.edu (A.J.)

**Keywords:** obesity, image classification, social media, twitter, food image

## Abstract

Obesity is a modern public health problem. Social media images can capture eating behavior and the potential implications to health, but research for identifying the healthiness level of the food image is relatively under-explored. This study presents a deep learning architecture that transfers features from a 152 residual layer network (ResNet) for predicting the level of healthiness of food images that were built using images from the Google images search engine gathered in 2020. Features learned from the ResNet 152 were transferred to a second network to train on the dataset. The trained SoftMax layer was stacked on top of the layers transferred from ResNet 152 to build our deep learning model. We then evaluate the performance of the model using Twitter images in order to better understand the generalizability of the methods. The results show that the model is able to predict the images into their respective classes, including Definitively Healthy, Healthy, Unhealthy and Definitively Unhealthy at an F1-score of 78.8%. This finding shows promising results for classifying social media images by healthiness, which could contribute to maintaining a balanced diet at the individual level and also understanding general food consumption trends of the public.

## 1. Introduction

### 1.1. Background

Obesity has more than doubled globally in the past three decades [1] and 1.9 billion adults worldwide are reported to be either overweight or obese [2]. Today, obesity is responsible for 5% of the total death count [1]. Should the current pace of obesity continue at this rate of increment, studies show that the life expectancy of obese people will be reduced by eight years [2]. Moreover, the global economic impact of obesity was estimated to be more than USD 2.0 trillion in 2016 [2].

**Figure 1:**
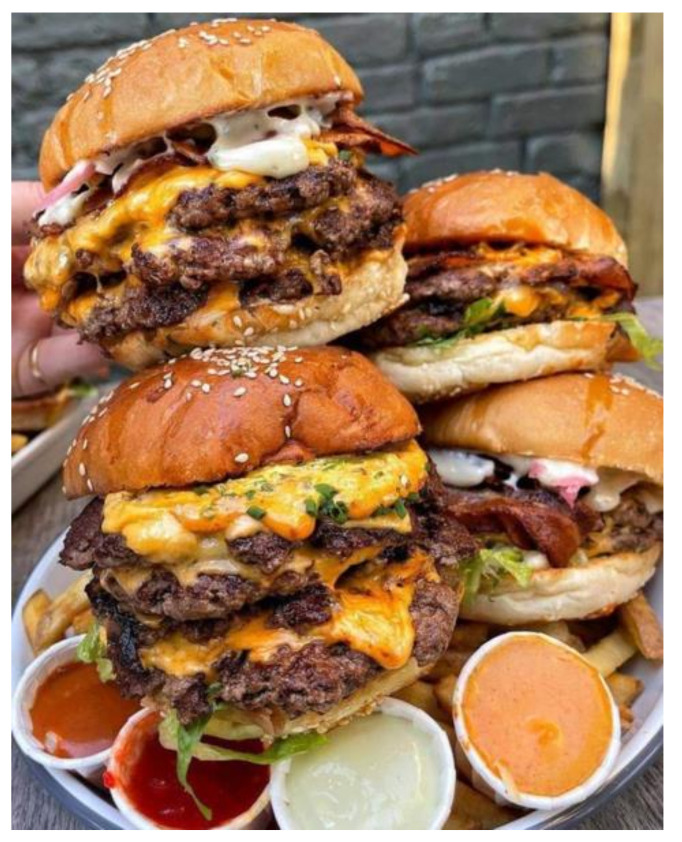
Example image of food from Twitter [5].

Sixty-five percent of American adults and 90% of young American adults now use social networking sites like Instagram and Twitter [3] to discuss everyday issues, socialize and share activities, such as dining on food. Food porn, the practice of sharing pictures of delicious, enticing foods has grown in popularity in recent years [4] (Figure 1).

In their study, Park et al. suggested that the general public’s everyday habits and beliefs concerning health can be observed using social media data [6]. In fact, food marketing on social media has been suggested to influence the intake of high-energy and low-nutrient foods such as fried foods and candy [7]. As several chronic illnesses and diseases such as cardiovascular disease, obesity, and cancer are associated with increased consumption of high caloric foods, this sort of influence can be critical when it comes to health outcomes [2]. Higher rates of chronic illnesses also lead to individual and country-level economic losses which majorly include the health care costs associated with obesity [2].

As of 2017, some 500 million tweets were posted daily on Twitter [8]. With the pervasive adoption of social media worldwide, this number can only be expected to grow. Because an increasing number of people’s lives and social interactions are publicly shared online, the breadth of social media data suggests that it can be a crucial tool in investigating lifestyle disorders [9].

As previously mentioned, food porn is a common practice on social media in which pictures of food are shared between users [8]. Simeone et al. demonstrated the power of social media to impact consumer behavior. They found that social networks homogenize food consumption choices by promoting particular foods [10]. Moreover, eating habits can be changed based on what users view on social media as well. This was demonstrated by Hawkins et al. in their laboratory study where they found that significant social media exposure to low energy dense food could nudge consumers to prefer low energy dense foods to high energy dense foods and vice versa [11].

Based on these findings, we could not only learn more about health-related behaviors from social media, but this information can be used to promote positive health outcomes. One method of studying group level food behaviors is by classifying healthy and unhealthy foods on social media. However, it should be noted that the captions of food pictures are not often descriptive of the food item. It is because of this, that food image classification is often used to classify the types of food shared on social media.

### 1.2. Literature Review

#### 1.2.1. Image Classification of Foods

Food image classification can prove to be a fairly difficult task, primarily due to the sheer variety of foods available nationwide. There are many characteristics of foods that may be appropriate features for classifying one group of foods, yet those same features may be ineffective when classifying another group of foods. As such, the process of feature engineering in this domain is closely linked with the success of the model.

Previously, feature extraction for image classification was conducted by hand. As can be imagined, this process was tedious and inevitably limited in the number of food classes recognized [12]. Researchers during this time commonly used Speed-Up-Robust Features (SURF) and textural features, such as local binary patterns (LBP) to assist in classification [12]. One study utilized a bag of features model that included Scale Invariant Feature Transform and HSV colors as features [13]. An SVM using these features achieved 78% classification accuracy [13].

As the popularity and accessibility of the framework increased, Deep Convolutional Neural Networks (DCNNs) began to be adopted as a means of extracting features to be ultimately used for classification. AlexNet, VGG-16 and GoogleNets are well known CNN architectures that can be used for deep feature extraction [12]. The process of image classification is many-fold. First, it must be determined whether or not a food item is present within the image. Singla et al. fine-tuned a GoogleNet pre-trained model with the Food-5K database for this task [14]. They managed to achieve a 99.2% accuracy score when distinguishing between food and non-food items.

Other than detecting the presence of food in an image, food item recognition is another common task that often utilizes CNNs. Farooq et al. used AlexNet features to recognize food items. The researchers fed features from convolutional layers to an SVM for classification [15,16]. They achieved 70.13% accuracy for 61 food groups. The accuracy score was raised to 94.01% for seven food groups [15]. An important note, though, is that the dataset used was the Pittsburg Fast-Food Image Dataset (PFID). Because this dataset was created in a controlled laboratory setting, the high accuracy rates should be taken as a grain of salt.

In a different study, researchers used the Instagram API to download a total of 808,964 Instagram posts using food related hashtags. For feature extraction, they used GIST as well as features from a pre-trained CNN. The CNN in this study was implemented using the MatConvNet toolbox and the 16-layer model was chosen. The researchers used ImageNet, a database built on the nouns found in WordNet, for their model. The output of their 16-layer CNN was a 4096-dimensional vector that the researchers believed would be ideal for generalizability. For classification, they used an SVM. Ultimately, by utilizing images and related discussions, the authors recognized food images at 70.00% accuracy [16].

In a Twitter related study, researchers developed a food detection approach that applied a CNN to recognize 10 food items. The researchers used local response normalization (LRN) as a hyperparameter for normalization after the pooling layers. For experimental purposes, they compared the CNN results to handcrafted feature generation methods. These included spatial pyramid matching (SPM), GIST and a SIFT-BoW-based method. A SVM was used as the classifier in each experiment [17]. Ultimately, they found that color features were dominant in food image recognition. For their final experiment, they aggregated a dataset collected from social media that contained a reasonable amount of noise (non-food item pictures). This model performed with an accuracy of 93.80% which is better than the baseline traditional SVM methods for which the accuracy was 89.70% [17].

#### 1.2.2. Utilizing Social Media to Understand Health Outcomes

Many previous studies have recognized the importance of social media images of food and its correlation to obesity. For example, in their study, [18] used FourSquare, a social media site that allows users to share their location within their social network, to map locations to Instagram photos of food. They then used the 2013 County Health Ratings (CHR), which contains county level obesity ratings, to demonstrate that unhealthy food hashtags are associated with the areas with high obesity rates [18].

In one Twitter-related study, the authors analyzed 210,000 tweets from US users to link their profiles to their tweeted dining experiences. They were then able to explore user interests, social connections and backgrounds based on the food that the users ate. For validation purposes, they associated the caloric intake of the foods tweeted with the user’s respective statewide obesity rate. The researchers obtained a Pearson correlation coefficient of 0.77 [19]. Once convinced that their approach had validity, the authors used this information to later build a model to predict county-wide obesity.

Twitter has been identified as a reliable source of information for the analysis of dietary patterns of individuals. It has demonstrated its ability to quantify the healthiness of food related tweets and related sentiments [20]. Regarding Twitter as a medium, the authors in [21] highlight the relevance of food on social media and the types of discussions surrounding food. Their study used food-related tweets to predict latent demographic factors, including obesity and diabetes rates, political learning, and author geography. This shows that the language of food alone is compelling [21].

One major limitation of these above-mentioned classification studies is the fact that manually labeling food images is time consuming and expensive. However, when manual labeling is not involved, experiments have performed poorly. Additionally, CNNs commonly face the issue of vanishing gradient descent. In this situation, weights are not changed a substantial amount, and as such, information is lost.

One way to remediate the latter issue is to use a ResNet. ResNets have grown in popularity due to their ability to both reduce the issue of vanishing gradient descent and reduce overall training time [12]. ResNet-152 is a deep residual CNN that has been pre-trained on ImageNet. It has a depth of up to 152 layers, which promotes higher accuracy scores [12]. ResNet-152 is constructed with residual connections that allow a gradient to pass through layers, bypassing an activation function. This is what decreases the effects of information loss [12].

More than 1.1 million obesity-related tweeted images were processed by a very deep Visual Geometry Group (VGG16) model, and it did not perform well. These results suggest the need for a fine-tuned image classification tool for social media images [6]. This is why we opted to adopt the ResNet-152 for training.

In their study, Vydiswaran et al. developed a four-point scale classifying a dataset of food keywords as Definitively Unhealthy, Unhealthy, Healthy and Definitively Healthy [20]. They achieved this by having a nutritionist use her expertise to rate a vocabulary of foods based on their healthiness level. They then organized the foods into the aforementioned groups based on these ratings [20]. We recognized the potential of this scale to be used in classification of food images as healthy or unhealthy. As such our aims of this study were three-fold. We wanted to demonstrate a process by which image datasets can be quickly constructed using the Google images search engine. Second, we wanted to build a transfer-learning based multi-class classifier to predict the healthiness of food based on Vydiswaran’s study. Lastly, we wanted to validate our classifier by applying it to social media food images from Twitter.

## 2. Materials and Methods

### 2.1. Overview

The explosion of image data on the Internet requires more sophisticated and robust models as well as algorithms to process images and related multimedia data [22]. Our work focuses on applying an image classifier to classify social media food images, specifically from Twitter, according to their healthiness. We fine tune the image classifier model, ResNet [23], for classifying social media images into healthiness level. We implement this model by transferring the features from the pre-trained ResNet model, which was initially trained on ImageNet dataset to the image classifier and training the classifier on images collected from the Google search engine. This research contributes as follows. First, we demonstrate the ability to build an image dataset without the labor-intensive process of manually labeling our images. Second, we build a transfer-learning based classifier to predict the healthiness of food. Third, we demonstrate the classifiers generalizability by applying it to food images from Twitter. We restricted our analysis to publicly available data.

### 2.2. Data Collection

To develop a healthiness food image dataset, we use a published list of sample food that is representative of definitively healthy (*n* = 5741), healthy (*n* = 5732), unhealthy (*n* = 5657), and definitively unhealthy (*n* = 6021) food images [20]. We then use the Google images search engine and crawl the search results. We manually verify the images and label each food item according to their respective categories: healthy, definitively healthy, unhealthy, and definitively unhealthy. These images were trained with the ResNet classifier by transferring the features from the ImageNet dataset. Each category contains ten types of food, and a total of more than 23,000 images were collected. We have also gathered the social media images from twitter using twitter API to examine external validity of our classifier.

### 2.3. Image Classifier

**Figure 2:**
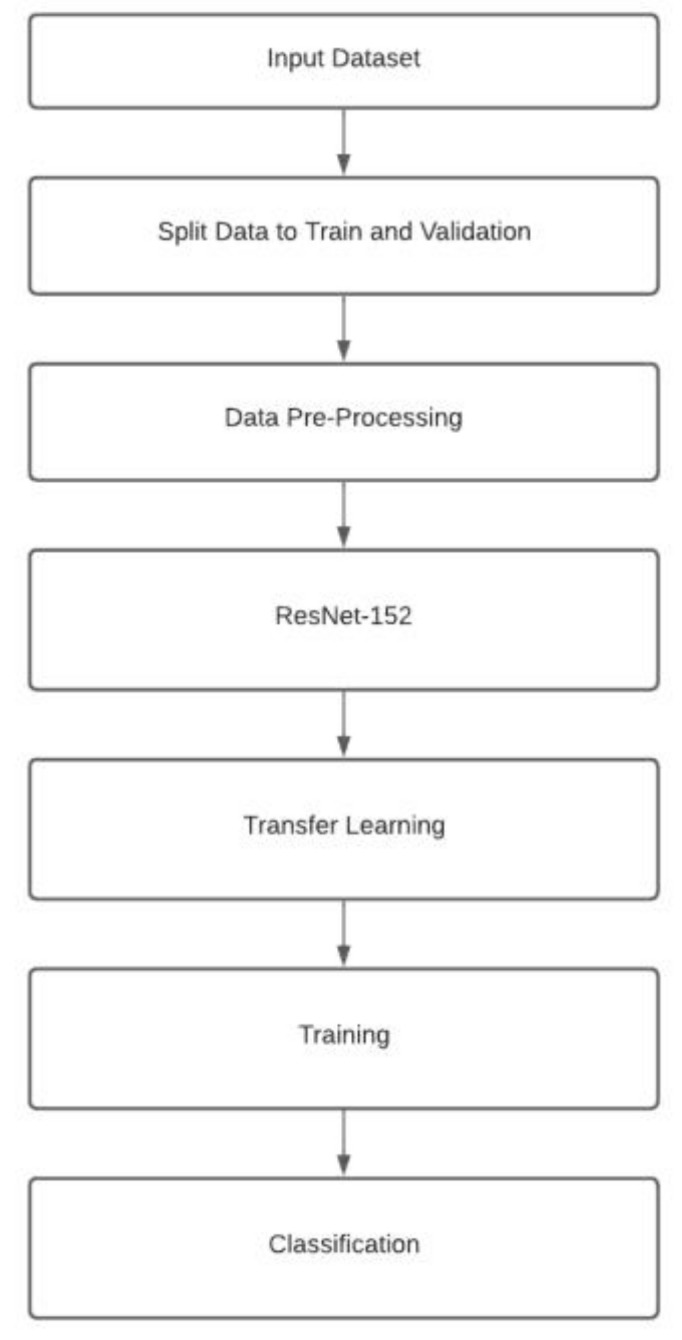
Overview of image processing step.

We used deep transfer learning implemented in ResNet-152 [4] to extract salient features of food images. ResNet152, a 152-layer residual net, is a deep network with a lower complexity than VGG nets [23], another popular image processing model based on ImageNet. The major steps of image processing are shown in Figure 2.

To classify food images as healthy, definitively healthy, unhealthy, and definitively unhealthy, we first center cropped each image to 224 × 224 following ImageNet standards to feed it to the deep neural network [24]. We experimented with different batch sizes and then decided on 128 bytes, which results in approximately 290 steps for each epoch. Then, features learned from ResNet-152 are transferred to a second network to train on the dataset [25]. The optimizer used for this classifier is Stochastic Gradient Descent (SGD) [26] due to its performance in our experiments. The trained SoftMax layer is stacked on top of the layers transferred from ResNet-152 to build our deep learning model, which is used to classify the test dataset [27]. The loss function used to find the loss for this model is categorical-cross entropy. We chose categorical cross entropy because it is well suited for multi-class classification.

These functions are directly imported from PyTorch packages, torch.nn, a module to help us in creating and training the neural network [28], and torch.optim, a package implementing various optimization algorithms [28]. Finally, we save the transferred layer’s parameters and train the ResNet model to classify each sample into its category. The experiment was carried out in an environment of Pytorch with CUDA 10.1 (Nvidia, Santa Clara, CA, USA) architecture on UBUNTU (Canonical, London, UK).

Lastly, we tested our classifier using an external dataset, Twitter image dataset. We processed 40 rounds of 10 food related twitter images, a total of 400, then manually evaluated the result. Overall accuracies are calculated by taking the average of the forty rounds of prediction.

## 3. Results

### 3.1. Training and Testing the Image Classifier

**Figure 3:**
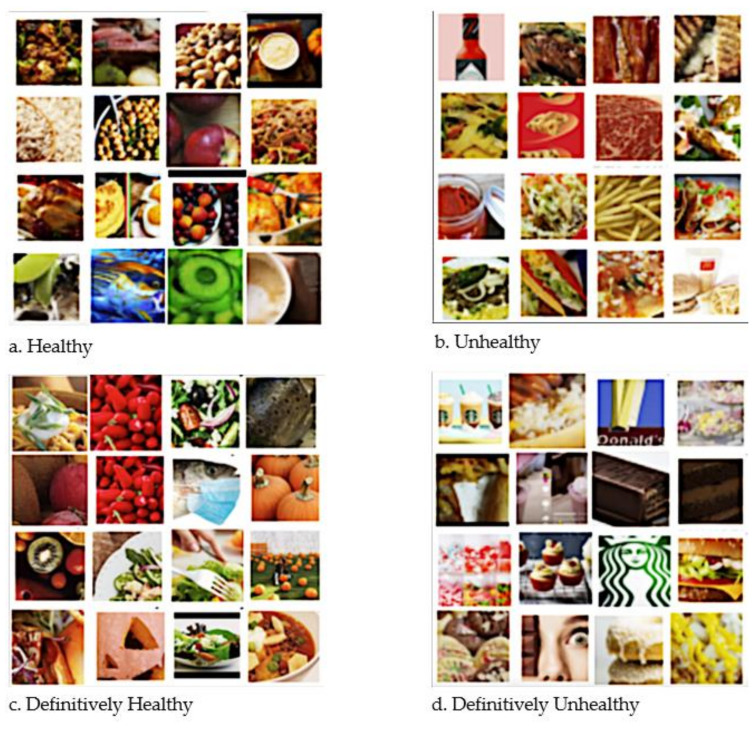
Example images that were predicted in each of the four classes.

We split the dataset 80–20 for train and validation. The validation accuracy is found to be 80.61%. We first trained the model on a different number of epochs up to 20 and decided the final number of epochs for training to be 15, to avoid overfitting of the training dataset. The trained model is tested on the validation images for all the categories of food. Figure 3 shows the prediction of food images in their respective classes. The bottom right contains the images of burgers, chocolate, and cake which are in the definitively unhealthy category. The bottom left image consists of the coffee, rice, turkey, and roasting which are in the healthy category. The top left image consists of the pumpkins, fruits, salad, and fish which are in the definitively healthy category. The top right has images of fries, taco, sauce, pizza, and grill which are in the unhealthy category according to previous literature [20].

### 3.2. External Validity: Testing on Twitter Dataset

**Figure 4:**
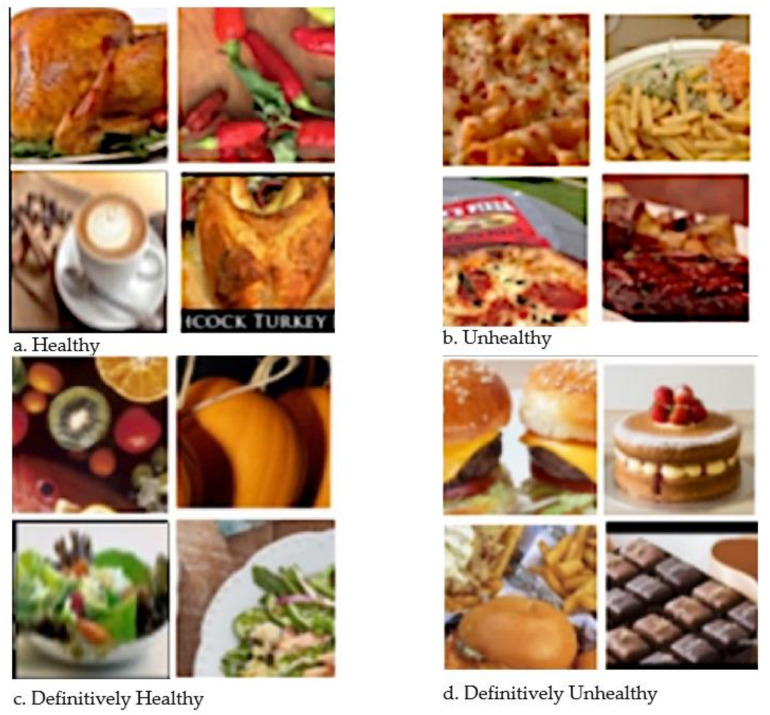
Example Twitter images that were predicted in the different classes.

The model is further tested on twitter images that were collected using the twitter API. We then manually selected images that contained food items from previous literature [20]. Examples of prediction are shown in Figure 4. The bottom right contains images of chocolate, cake and burger which are in the definitively unhealthy category. The bottom left consists of pumpkin, fruits and salad which are in the definitively healthy category. The top right has the images of turkey, chili and coffee which are in the healthy food category. The top left shows pizza, fries and roasting images which are unhealthy food items.

We also assess the performance of our classifier at the individual category (Table 1). TP, TN, FP and FN stand for true positive, true negative, false positive and false negative, respectively. TP refers to the number of predictions in which the classifier correctly predicts as positive. TN refers to the number of times the classifier correctly predicts as negative. The number of predictions in which the classifier incorrectly predicts the negative class as positive is referred to as FP. The number of predictions in which the classifier wrongly predicts the positive class as negative is referred to as FN.

The percentage of positive identifications which were actually correct (i.e., precision) was 72.13 in case of healthy, 68.42 in case of unhealthy, 78.57 in case of definitively healthy and 76.36 in case of definitively unhealthy. The proportion of correctly identified positives (i.e., recall) was 88.00 in case of healthy, 78.00 in case of unhealthy, 88.00 in case of definitively healthy and 84.00 in case of definitively unhealthy. The percentage of all the correctly identified cases (i.e., accuracy) is 77.00 in case of Healthy class, 71.00 in case of unhealthy, 82.00 in case of definitively healthy and 79.00 in case of definitively unhealthy. F1 Score for healthy class is 79.27%, unhealthy class is 72.90%, definitively healthy class is 83.01% and definitively unhealthy class is 79.99%. The overall F1 score was determined to be 78.78%.

### 3.3. Error Analysis

We further analyzed errors from each of the classes. Table 2 shows how errors for each classification class were classified. The most frequent inaccurate prediction came from the degree of healthiness. For example, as our classifier incorrectly predicted the healthy with definitively healthy as well as unhealthy with definitively unhealthy classes. The result of FN for Healthy 4 images out of 6 images were predicted as definitively healthy. Similarly, for FN of unhealthy class, out of 11 images, 7 images were inaccurately classified as definitively unhealthy. For definitively unhealthy, out of 8 FN, 4 of them were incorrectly predicted as unhealthy. In the case of definitively healthy class, 4 out of 6 FN were inaccurately classified as healthy. With FP, out of 17 times where the prediction was incorrectly classified as healthy, there were definitively healthy 9 images. Out of 18 times where the prediction was incorrectly predicted as unhealthy, there were 8 definitively unhealthy images. Out of 12 times where the prediction was inaccurately classified as definitively healthy, there were 6 healthy. Out of 13 times where the prediction was inaccurately classified as Definitively Unhealthy, there were 7 unhealthy images.

After manual investigation, we also believe that the classifier is mixing up baking (i.e., healthy) with cake (i.e., definitively unhealthy) and detects definitively unhealthy as healthy. The combined number of Twitter test images for cake and baking were 25, out of which 5 baking images were predicted as definitively unhealthy, and 6 cake images were classified as healthy. Table 3 shows the number of images and the number of false positives and false negatives for the cake and baking food items. This is partially due to how the training dataset was collected.

In our training dataset, we found 254 cake images, as a part of cake recipe, in 996 baking images.

## 4. Discussion

### 4.1. Principle Findings

The main contributions of our research are the following: (1) demonstrating a process to quickly construct an image dataset using the Google images search engine to reduce a labor-intensive process of image labeling, (2) building transfer learning based multi-class classifier to predict healthiness of food and (3) applying the classifier on social media images from twitter to understand classifier’s real-world application.

Supervised learning needs labeled datasets for the classification. Studies typically use pre-existing food image datasets available on public repositories [29,30] or build new datasets with manual labeling which is an expensive and time-consuming task [31,32]. For training the residual network, we have crawled images from Google images search results. We were able to construct a labeled dataset in a relatively short period of time. However, we manually went through all of the images to verify and ensure the quality of the dataset and classifier.

To test the performance with real world social media data, we built a testing dataset containing real-world food images from twitter. To test against human judgement, we manually evaluated 400 Twitter images. The model was able to classify the images into the healthiness classes at an F1 score of 78.78%. The most frequent inaccurate prediction came from identifying the degree of healthiness of food images as the classifier inaccurately classified as healthy for definitively healthy or as unhealthy for definitively unhealthy food images. The performance of our image classifier is better than the F1 scores reported by other deep CNN approaches that are applied in public health informatics research [6,16,29,33]. We were able to improve the performance by transfer features from a neural network with a greater number of layers, ResNet 152. A few other approaches achieved better than 80.00% F1 scores; however, their objectives were relatively a simple task of binary identification of food image [14,34].

### 4.2. Public Health Implications

Twitter has been demonstrated as a good data source for tracking public health issues like influenza [35,36] seasonal allergies [36], cholera outbreaks [37], mental health [37,38,39] and behaviors like excessive and unhealthy food consumption [6]. However, these studies mainly used text as the only data source. Although a growing number of Twitter users are sending out images, image analysis has not been the main focus of most previous public health studies using Twitter, in part due to the difficulty of accurately processing images [6,19,35]. Although images are often accompanied by captions, oftentimes these captions are not descriptive of the images themselves [16]. As such, there is a missed opportunity for collecting more descriptive social media data. Our study focused on processing food image data specifically. There are many potential benefits of fully utilizing social media data’s multimodal nature. Processing images and classifying them as healthy or unhealthy provides additional data points for not only public health researchers and practitioners, but individuals themselves. This technology could be used as a method of food logging. Food logging is a common technique used by dieticians and nutritionists in which individuals keep track of the foods that they consume for later review and reflection. In this case, users could passively keep track of the foods that they consume for later inspection.

Likewise, information gathered from our study could be used to improve healthy literacy in certain populations. Health literacy has been defined as the overall ability for one to take control of their health [40]. Health literacy is a well-documented public health problem, particularly among lower socioeconomic backgrounds [41], who are at an increased risk of being overweight or obese [1]. Furthermore, some public health information may be too difficult for the general public [42]. Thus, by automatically processing to inform users of the food’s healthiness level and maintaining an easily manageable communication channel could improve health literacy regarding food consumption. On a broader scale, our research could help those who work in the public health domain to understand public consumption of foods and food behaviors. Due to social media’s transient nature, these researchers and clinicians could also get a better understanding of how dietary patterns change over time. This could provide some insight into potential triggers for unhealthy food consumption, identifying food deserts and even understanding marketing materials that might inspire unhealthy food behaviors.

The ability to correct negative dietary influences could potentially lead to better health outcomes as a nation. This would be crucial as obesity has grown in the United States two-fold for the last three decades. Findings of this research can enhance consumer-generated text processing as well. Processing consumer-generated text, especially a short Twitter text, is complex, due to issues like acronyms, abbreviations, slang, and topic drift [43,44]. Image processing research with textual description could bridge textual and visual information [9,40] and further improve the performance of processing consumer-generated text.

### 4.3. Limitations and Future Direction

A shortcoming of our classifier is that the model may be slightly overfitting, which could explain the lower performance when using the real-world Twitter images. Social media images could also have background noise and occlusion. We did not segregate images to remove irrelevant items in the training dataset, which could improve the results when applying to social media images. Additionally, our classifier only accounts for one individual food item, not different foods grouped together into a meal. For example, it is unable to label a meal of a burger and salad as either healthy or unhealthy. Future studies could perform image segmentation first in order to identify different types of food and then classify those food items based on their overall healthiness.

We can further improve the real-world application of our classifier by adding more food items to the dataset in their respective categories to cover a wider variety of food images. In addition, our training dataset could contain inaccurately categorized images, as shown in baking and cake images, even though the images were manually verified. The next step for this study is to further refine the dataset and test for different social media images on networks like Instagram as they can also contain the food images shared by the users.

Specific to obesity research, future study can further analyze the healthiness of social media food images in correlation with obesity rate of specific regions. This will further extend our knowledge on how social media is impacting food consumption in comparison to other known factors related to obesity pandemic, such as environment.

## 5. Conclusions

This research examines a relatively simple approach to build a healthiness image classifier by leveraging transfer learning and image dataset collected using the Google images search engine. To assess the reliability and efficacy of our approach, we perform experiments using an external social media dataset. Results show that transfer learning for food image classification performed at an accuracy of 80.61% on the training dataset and achieved an F1 score of 78.78% when processing Twitter images. While there are a few improvements that can be made to this model, it already shows potential in improving health outcomes concerning obesity and the consumption of unhealthy foods. Our algorithm could potentially be used to improve healthy literacy and assist public health researchers and practitioners in understanding behaviors contributing to unhealthy food consumption.

## Figures and Tables

**Table 1:** Performance of the image classifier on Twitter datasets.

Class	TP	FN	TN	FP	Precision	Recall	Accuracy	F1 Score
Healthy	44	6	33	17	72.13	88.00	77.00	79.27
Unhealthy	39	11	32	18	68.42	78.00	71.00	72.90
Definitively Healthy	44	6	38	12	78.57	88.00	82.00	83.01
Definitively Unhealthy	42	8	37	13	76.36	84.00	79.00	79.99
Overall	169	31	140	60	73.79	84.50	77.25	78.78

**Table 2:** Error analysis using Twitter datasets.

Class	Predicted Healthy		Predicted Unhealthy		Predicted Definitively Unhealthy		Predicted Definitively Healthy	
	FN	FP	FN	FP	FN	FP	FN	FP
Healthy	–	–	–	4	2	4	4	9
Unhealthy	3	4	–	–	7	8	1	6
Definitely Healthy	4	6	1	3	1	3	–	–
Definitely Unhealthy	2	3	4	7	–	–	1	3

**Table 3:** False positives and false negatives for the cake and baking.

Food Items	Predicted as Definitively Unhealthy	Predicted as Healthy
Cake (Definitely unhealthy)	7	6
Baking (Healthy)	5	7

## Data Availability

Not applicable.
